# Activity and social interactions in a wide-ranging specialist scavenger, the Tasmanian devil (*Sarcophilus harrisii*), revealed by animal-borne video collars

**DOI:** 10.1371/journal.pone.0230216

**Published:** 2020-03-23

**Authors:** Georgina E. Andersen, Hugh W. McGregor, Christopher N. Johnson, Menna E. Jones

**Affiliations:** 1 School of Natural Sciences, University of Tasmania, Hobart, Australia; 2 School of Natural Sciences and Australian Research Council Centre of Excellence for Australian Biodiversity and Heritage, University of Tasmania, Hobart, Australia; Universidad Austral de Chile, CHILE

## Abstract

Observing animals directly in the field provides the most accurate understanding of animal behaviour and resource selection. However, making prolonged observation of undisturbed animals is difficult or impossible for many species. To overcome this problem for the Tasmanian devil (*Sarcophilus harrisii*), a cryptic and nocturnal carnivore, we developed animal-borne video collars to investigate activity patterns, foraging behaviour and social interactions. We collected 173 hours of footage from 13 individual devils between 2013 and 2017. Devils were active mostly at night, and resting was the most common behaviour in all diel periods. Devils spent more time scavenging than hunting and exhibited opportunistic and flexible foraging behaviours. Scavenging occurred mostly in natural vegetation but also in anthropogenic vegetation and linear features (roads and fence lines). Scavenging frequency was inversely incremental with size e.g. small carcasses were scavenged most frequently. Agonistic interactions with conspecifics occurred most often when devils were traveling but also occurred over carcasses or dens. Interactions generally involved vocalisations and brief chases without physical contact. Our results highlight the importance of devils as a scavenger in the Tasmanian ecosystem, not just of large carcasses for which devils are well known but in cleaning up small items of carrion in the bush. Our results also show the complex nature of intraspecific interactions, revealing greater detail on the context in which interactions occur. In addition, this study demonstrates the benefits of using animal-borne imaging in quantifying behaviour of elusive, nocturnal carnivores not previously seen using conventional field methods.

## Introduction

Carnivores can have strong top-down influence in ecosystems, altering the abundance and behaviour of smaller predators and prey, with sometimes cascading effects onto vegetation and ecosystem processes [1–3]. Documenting the behaviour of carnivores in their natural habitat to understand their foraging and social ecology is often difficult, especially for highly mobile nocturnal species that may change their behaviour in response to the presence of an observer. Recent advances in camera technology have made it possible to use animal-borne video collars to sample such fine-scale behaviours [4].

Tasmanian devils (*Sarcophilus harrisii*; ‘devils’ hereafter) are the largest extant marsupial carnivore and are endemic to the island state of Tasmania, Australia. Devils are the top mammalian predator in Tasmanian ecosystems and their severe widespread population decline in the last 25 years due to a novel transmissible cancer (devil facial tumour disease) [5] has triggered trophic cascades leading to mesopredator release of invasive feral cats (*Felis catus*) and rodents [6, 7]. Devils are difficult to observe in the wild because they are nocturnal [8], cryptic and avoid humans. Studies of the natural behaviour of devils have been limited to techniques which do not reveal many of the intricacies of foraging behaviour and social interactions. Direct behavioural observations are limited to those around carcasses of prey [9–11] and incidental observations near roads at night [12]. Inferences about foraging and social behaviour are made from tracking studies, including spool-and-line tracking [13], and VHF, GPS or proximity-sensing radio collars [14–17].

Devils have the skeletal features of pounce-pursuit predators that are capable of short fast pursuits [18] and they appear to forage with a moving search [19]. Knowledge of their diet, which comes from analyses of scat and stomach contents, shows devils to be generalist foragers that predominately consume medium to larger-sized mammals [20–22]. Hunting is infrequently observed in wild devils but demonstrates that devils can kill animals larger than themselves (e.g., Bennett’s wallabies (*Macropus rufogriseus*) and bare-nosed wombats (*Vombatus ursinus*)) using a crushing bite to the head or neck region [23]. Devils are also one of a few mammalian carnivores, globally, that have morphological adaptations for eating bone, the other species being the hyaenas [24, 25]. Devils and hyaenas are an example of ecomorphological convergence between marsupial and placental carnivore guilds. The relative importance of scavenging and hunting in the diet of hyaenas was established 50 years ago [26] but the extent to which devils scavenge *versus* hunt to kill prey has not been quantified.

Devils are a solitary carnivore that have overlapping home ranges (2531ha) [27], and interact agonistically around prey carcasses and during mating [9–11]. Knowledge of social contact and biting behaviours relevant to disease transmission is crucial for understanding disease spread and epidemic outcome, and for informing disease management options. Proximity-sensing radio collars have been used to investigate interactions amongst devils [14, 17] but they do not provide any information on location or whether the interactions were agonistic, although the latter can be inferred when coupled with examination of bite wounds from collared animals that are regularly trapped [17]. Video collars can provide a more detailed understanding of the location and nature of social interactions among devils.

This is the first study to use animal-borne video collars to investigate the natural behaviour of wild devils. The study site in northwest Tasmania, Australia, was not affected by DFTD at the time and so provides a base-line of natural devil behaviour unaffected by the DFTD epidemic. We aim to: (1) quantify the relative contribution of predation vs. scavenging to the diet; (2) document hunting behaviour and assess the frequency of hunting and killing events; (3) quantify the frequency and intensity of intraspecific interactions with the expectation that the majority of intraspecific interactions will occur over carcasses; (4) evaluate the usefulness of video collars in better understanding devil behaviour.

## Materials and methods

### Ethics statement

This study was carried out in accordance with the University of Tasmania Animal Ethics Committee Permit #A12361 and A15221with permission from the Tasmanian Department of Primary Industries, Parks, Water and Environment (DPIPWE) under scientific permits TFA17003, TFA16115 and TFA15239.

### Study area

**Fig 1:**
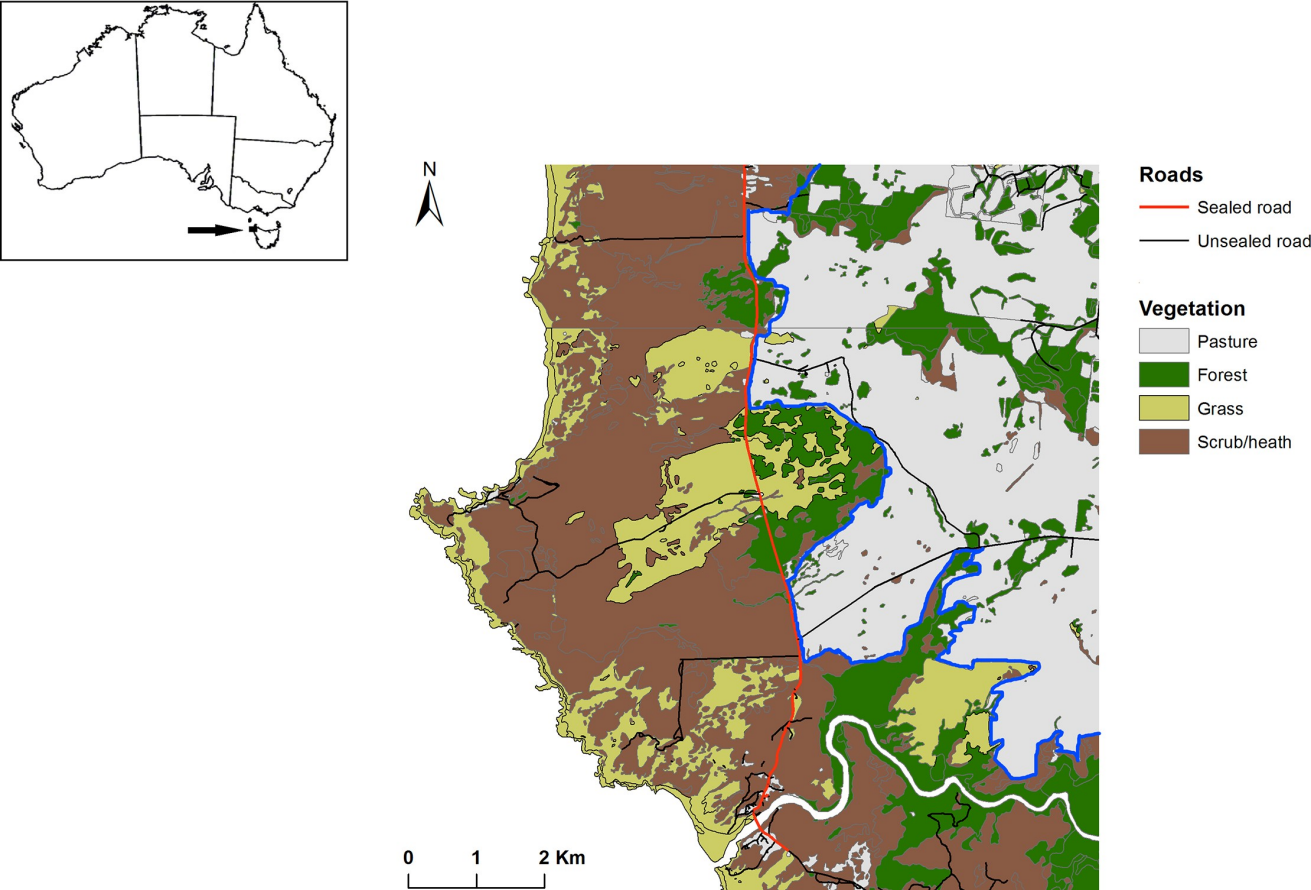
Location of the study area in northwest Tasmania, Australia. The major vegetation types and roads are displayed. The Conservation Area is located to the west and south of the blue line.

The study was conducted in the far northwest of the island state of Tasmania, Australia. The study area comprised part of the Arthur-Pieman Conservation Area (41.04°S, 144.66°E) in the west, bounded by the Indian Ocean, and adjacent private livestock properties to the east (Fig 1). The two land tenures are incompletely separated by a sealed road that runs north—south through the middle of the study area. Native vegetation to the west of this road consists of coastal scrub/heath (*Leptospermum scoparium*, *Acacia longifolia* and *Melaleuca squarrosa)* and moorland (*Gymnoschoenus sphaerocephalus*). Vegetation to the east of the sealed road consists of a mosaic of eucalypt forest dominated by *Eucalyptus obliqua* and *E*. *nitida* and exotic pasture grazed by cattle (Fig 1). The only houses at the study site are the two farms to the east of the road and fewer than 20 houses on the coast to the west of the road. Devils use all of the vegetation and land tenure types on the study area, which influence the presence and abundance of prey and the foraging activity of devils [16].The climate is temperate, with monthly mean temperatures ranging from 9.4–16.1°C, and mean annual rainfall of 1069mm [28].

The study area was ahead of the disease front and devils remained unaffected by DFTD throughout the study period. This was intentional as base-line information on the nature of intraspecific contacts in healthy devils is needed before studying how the disease changes these. In diseased populations, prevalence is 30–50% among adults and population age-structure is strongly skewed to subadults with nearly all adults contracting DFTD and dying within a year of reaching sexual maturity [29, 30]. The devil population at the study site was at relatively high abundance (4.6 devils per km^2^ (Potts et al., Unpublished data)).

### Video collars

**Fig 2:**
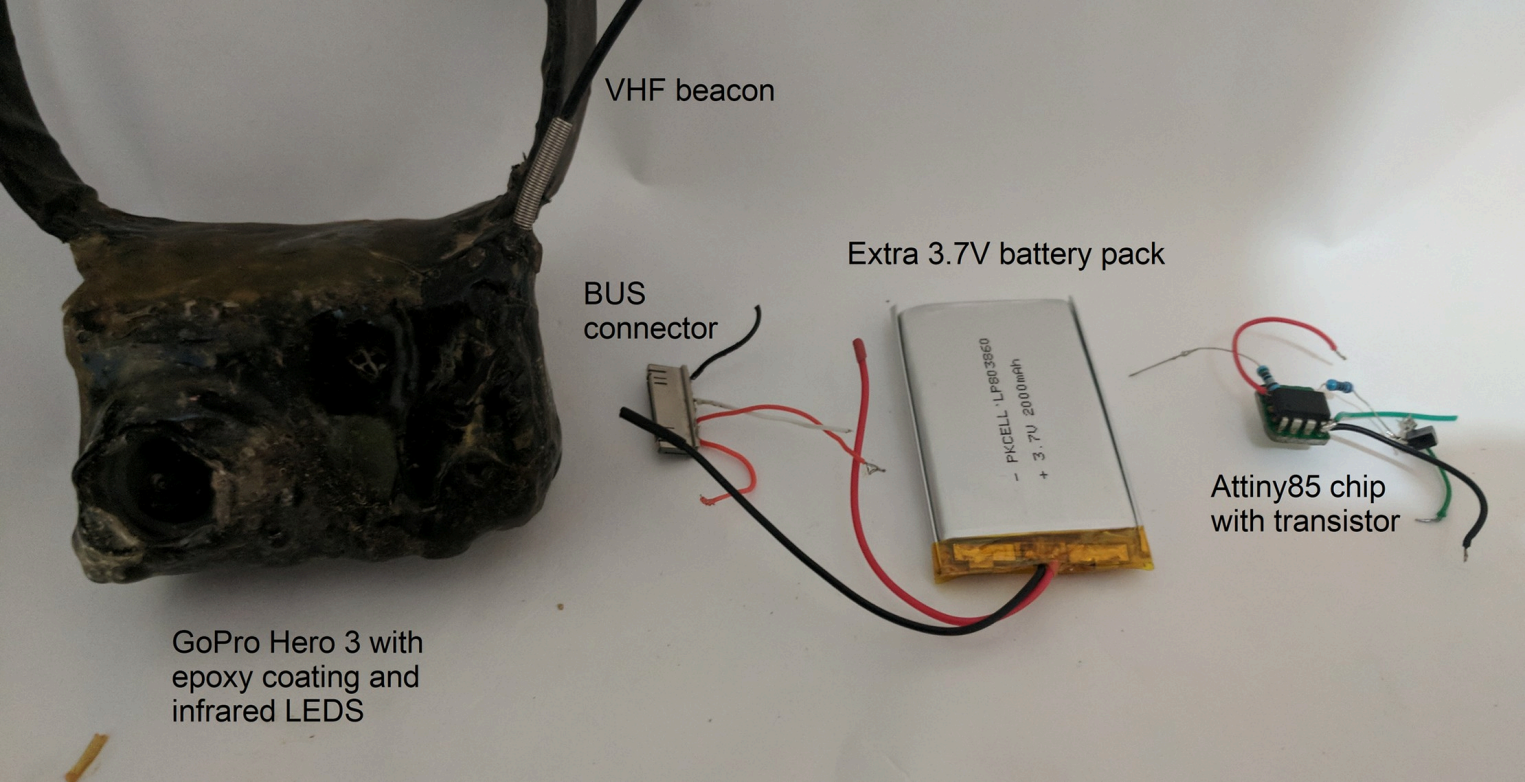
Image of camera set up.

Video collars were created by modifying GoPro Hero 3 White video cameras (GoPro Inc, San Mateo, California, USA). Modification involved removing the camera’s infra-red filter, adjusting the focal range to between 1-100cm, adding either four or six infra-red LEDs (920nm) adjacent to the lens, placing a scratch-resistant glass cover over the lens, soldering external wires onto the battery terminals, and connecting these wires to external batteries. Collars of different weights were constructed, where a greater volume of batteries were placed on collars for larger devils, ranging from one to three 1100mha lithium-ion batteries, or two 18500 batteries in parallel. Collars were developed and improved throughout the study. For some collars, delay timers were created using Ardunio Atiny85 chips (ATMEL, San Jose, California, USA) connected to the camera’s controls via the BUS port (pins 1,12, 23 and 24), and were set to start recording 11 hours after they were activated. Every video-collar had a separate VHF transmitter installed in the package (Sirtrack Ltd, Havelock, New Zealand), and a nitrile collar coated in black heat-shrink tubing. The assembly was hand-coated in two layers of epoxy resin (SC651, Solid Solutions, Bentleigh East, Australia). Collars were fastened with corrodible links which degrade over time and would eventually allow the collar to fall off if the animal was not re-trapped [31]. The finished video collars could record between 2–12 hours of footage, weighed between 100-220g, and had maximum dimensions of 65 mm × 50 mm × 45 mm (Fig 2).

### Deployments

Video collars were deployed 45 times on 16 different adults (5 females and 11 males) between September 2013 and February 2017 across three seasons. Deployments were timed to sample three seasons that include significant life-history events: summer (January-February, immediately before the mating season and when juveniles were being weaned and dispersing); autumn/winter (May-July when females were carrying pouch young that ranged during this time from small to fully furred, with an increasing energetic burden); and spring (September-November when young are left in a den and lactating females return to provision them). Adult males were tracked throughout the three seasons, whereas females were only tracked in summer and spring when they were lactating or weaning their young.

Devils were trapped in custom built pipe traps (diameter 315 mm x length 875 mm, constructed from solid PVC pipe; N. Mooney and D. Ralph, unpublished data) baited with meat. Nearly all of the devils on the study site (n = 142) were already microchipped as part of a long term study on devil movement [16, 27]. Only devils that have been trapped several times before were fitted with a camera, to ensure a high probability of recapture for collar retrieval. Several males were trapped frequently, which led to us collaring more males than females. Animals were not sedated and were released immediately following fitting of collars. Devils that were fitted with a timer collar or with a video collar that was turned on during the day were released from traps early in the morning. To fit devils with a video collar that was turned on during the night, traps were set at dusk and checked a few hours later, and the devils released immediately after they were fitted with a collar. Eight devils were released with the video collar turned on during the day, 12 were released in the night, and 10 animals were fitted with timer collars that turned the video on 11 hours after release. The different types of collar were randomly deployed on devils. As collars recorded footage for a maximum of 12 hours, devils were re-trapped as soon as possible thereafter and the collars removed.

### Data handling and analysis

#### Data handling

First, we reviewed all footage and recorded the start and finish time of all behaviour states observed (moving, scavenging, hunting, interacting with other devils, drinking and resting). Scavenging was defined as when a devil was feeding on an animal that was already dead when the devil found it. Hunting was defined for the purposes of this paper as active pursuit and capture of live prey, regardless of whether the prey was killed or not, although devils are probably actively hunting every time they are moving. An interaction was defined as the presence of another devil in close proximity to the video-collared devil, or if vocalisations could be heard. Resting included sleep, grooming and digging (as devils dug only while in their burrows). To give context to later analyses and the timing of certain behaviours, we calculated the average proportions of time the devils spent in each state and compared this across three periods of the diel cycle: midnight to sunrise, sunrise to sunset and sunset to midnight. These time periods were based on phases of devil activity determined from GPS data at this study area [27], which showed that most devils became active at sunset, movement rates gradually reduced after midnight, and most devils were stationary by sunrise. The first hour of footage following the release of a devil from the trap was considered likely to be atypical and was excluded.

#### Scavenging and hunting

The size of scavenged items was categorised as very large (>1m in length), large (~1m), medium (~0.5m) or small (<0.5m). The size of items was estimated from the footage in relation to the devils head or based on prior knowledge of the size of the object in question. Devils can use native vegetation, fence lines, pasture and roads for foraging [16]. Seabird feathers and fur seal fur have been found in devil scats [21], which suggests that devil patrol beaches in search of items to hunt or scavenge. It was relevant and interesting, therefore, to quantify the frequency of scavenging in each of these locations. For scavenging events, the item was identified where possible, and its size, location (natural vegetation, pasture, road, fence line, beach, near a house) and the duration (seconds) of time the devil spent eating the item recorded.

For hunting events, the prey item and the devil’s behaviour during the different components of the predatory sequence were recorded, the latter including duration and movement pattern of the pursuit, the location on the body of the prey where the devil grasped the prey and how the devil killed the prey.

#### Intraspecific interactions

Every intraspecific interaction and scavenging and predatory event was examined in detail. For interactions, the context of the location (near or in a den, traveling, or at a carcass), duration of interaction (seconds), bite duration (seconds) and type of vocalisation was recorded. To examine factors affecting the rate of interactions between devils, we considered every minute of footage as a datapoint. For each minute, we recorded context of the location (near or in a den, traveling, or at a carcass), diel period (within two hours of sunrise/sunset, day or night), and whether within 30 minutes of release (post release). We then used generalized linear mixed-effect binomial models (GLMMs) in the package ‘nlme’ [32] in R, version 3.1.3 [33] to compare all combinations of these variables and a null model (seven models in total). Whether an interaction occurred or not in each minute was used as a binary response variable in all models. As results could have been correlated between individuals and deployments, both were added as a random effect in all models. We then compared these seven models within an information theoretical framework, and selected the most parsimonious model based on the lowest Akaike Information Criterion (AIC) score [34].

## Results

A total of 173 hours and 48 minutes of footage were collected, from 30 deployments on 13 individuals (3 females and 10 males) between 2013 and 2017 (Table 1). Fifteen collars failed to record footage, either due to camera malfunctions or the waterproof housing being chewed and destroyed by other devils during agonistic encounters. As we excluded the first hour after release of a devil, 143 hours and 48 minutes of footage were analysed. Most footage of devils being activate (i.e. not resting) was obtained at night, but even so, resting behaviours (sleep, rest, and self-grooming) were still the most frequent activities in all diel periods (Table 2). Scavenging was observed on average 5 to 7% of each devil’s night of activity, while short spans of time were spent hunting, interacting with other devils, or drinking (Table 2).

**Table 1:** Video collar deployment data. Summary data for each individual devil.

ID	Sex	Date	Time video started recording	Video duration (hh:mm:ss)
Agamemnon1	Male	16/09/13	07:00pm	03:12:31
Larissa1	Female	09/10/14	07:00pm	02:20:24
Sansa1	Female	09/10/14	08:00am	04:31:11
Larissa2	Female	17/10/14	11:00pm	02:25:57
Pavo1	Male	20/10/14	01:00am	04:25:01
Larissa3	Female	26/10/14	07:40am	01:58:49
Pavo2	Male	26/10/14	06:40pm	05:59:12
Linus1	Male	30/10/14	07:00pm	02:30:51
Sansa2	Female	30/10/14	11:50pm	06:23:12
Pavo3	Male	04/11/14	08:35pm	12:23:46
Theia1	Female	14/01/16	07:30am	03:20:19
Freddy1	Male	14/05/16	08:00pm	10:53:10
Cadoc1	Male	17/05/16	09:00am	01:16:38
Chimp1	Male	14/05/16	06:00pm	01:33:50
Prince1	Male	22/05/16	08:00am	01:10:44
Tofino1	Male	13/06/16	09:00pm	07:09:14
Tofino2	Male	15/06/16	09:00pm	10:35:30
Chimp2	Male	20/06/16	01:00pm	10:36:07
Tofino3	Male	20/06/16	09:00pm	06:03:12
Tofino4	Male	23/06/16	09:00pm	12:01:10
Cashy1	Male	07/07/16	02:00pm	10:38:20
Theia2	Female	04/01/17	07:00am	01:26:32
Cadoc2	Male	05/01/17	07:10pm	10:49:18
Tofino5	Male	05/01/17	07:20pm	02:27:28
Theia3	Female	05/01/17	08:45am	04:23:45
Theia4	Female	07/01/17	02:30am	05:55:00
Cadoc3	Male	10/01/17	09:00am	05:58:00
Tofino6	Male	01/02/17	07:00pm	10:37:32
Tofino7	Male	05/02/17	07:30pm	09:52:48
Big Scare1	Male	10/01/17	06:00pm	04:01:44

**Table 2:** The percent of time recorded of devils witnessed conducting various behaviours (including standard errors). As these are averages, not totals, figures do not equal 100%. Data have not been analysed, and this is principally descriptive.

	Midnight–Sunrise		Sunrise–Sunset		Sunset–Midnight	
	Average %	SE	Average %	SE	Average %	SE
Resting	62.30	2.80	84.80	1.90	67.10	2.50
Drinking	0.30	0.01	0.10	0.01	0.30	0.01
Hunting	0	0	0.02	0	0.01	0
Scavenging	7.20	0.40	0.70	0.05	5.00	0.20
Interacting	0.10	0.01	0.08	0.01	0.20	0.02
Moving	30.30	1.00	13.30	0.70	27.40	0.70

### Scavenging

Scavenging events were recorded 84 times, of which the items scavenged could be identified in 19 cases. Of these, 60 occurred in natural vegetation, 16 in pasture, three on a beach, two on a road, two near a house and one along a fence line. Scavenging frequency was inversely incremental with size e.g. small carcasses were scavenged most frequently (Table 3). Identifiable items consisted of cow (*Bos taurus*) (n = 4), macropod (either Tasmanian pademelon *Thylogale billardierii* or Bennett’s wallaby *Macropus rufogriseus*) (n = 6), brushtail possum (*Trichosurus vulpecula)* (n = 1), snake (n = 1), bird (n = 3), Tasmanian native hen (*Tribonyx mortierii*) egg (n = 1) and devil scats (n = 3).

**Table 3:** Number (n), average duration, and the rate of scavenging events by prey size per hour that devils were active. Rate is for individual events. Time spent scavenging per hour is presented in Table 2.

	Total (n)	Average duration (mm:ss)	Rate/ hr of activity
Very large	4	22:32	0.05
Large	7	21:44	0.09
Medium	10	08:37	0.13
Small	63	02:29	0.83
**Total**	**84**	**55:22**	**1.10**

### Hunting

We recorded two hunting events, one on a medium-sized mammal (either a European rabbit *Oryctolagus cuniculus* or a juvenile Tasmanian pademelon), and one on a bird or rodent. In the first instance, a 10.6 kg male devil was running at a constant speed through an open livestock paddock during the night, when he paused for a couple of seconds and then ran rapidly towards a medium-sized mammal. The prey animal did not move and the devil grabbed it with his teeth on the front left forearm. The animal struggled for nine seconds in the jaws of the devil before it escaped. The devil did not attempt to pursue it further but stood growling and snorting. At the time of the attack, there was a full moon directly in front of the devil at the point of attack, which may have affected the ability of the devil to grab the prey correctly. Three days later the same devil preyed on either a bird or rodent that it appeared to encounter opportunistically on the forest floor just after sunrise. The animal made a few short high-pitched cries before the devil quickly consumed it. On both occasions, the devil exhibited an opportunistic search pattern before encountering the prey and there was no prolonged chase or stalking behaviour.

### Intraspecific interactions

Intraspecific interactions were recorded 33 times and lasted on average 30 seconds (range: 1–85 seconds). All interactions were aggressive and involved growling and/or snorting in all but two events. Most interactions occurred when the collared devil was traveling and encountered another devil (n = 18), with fewer interactions recorded either near or in a den (n = 6) or at a carcass (n = 9). In all cases of interactions at dens, the devil in initial possession of the den won and displaced the other devil. Physical contact and biting occurred eight times (twice in den, twice over a carcass and four times when the devil was traveling), and lasted for less than two seconds except for two biting events, which lasted for nine and 55 seconds, respectively. Physical contact and biting occurred in only 24% of encounters. Bites were delivered only to the face during the nine-second interaction, whereas bites were delivered to the face and body during the 55-second interaction.

When we modelled interaction probability per unit of time, the most parsimonious model (i.e. with the lowest AIC value, Table 4) included only devil location, and suggested that the odds of a devil interacting per minute increased 6-fold when a devil was traveling compared to a den (95%CI = 2.4–16.7, z = -3.77, P < 0.001), then a further 2.7-fold if at a carcass (95%CI = 1.1–6.4, z = 2.2, P = 0.023).

**Table 4:** Results of AIC model selection between different variables affecting a devils probability of detection with another devil, including whether traveling, in a den, or by a carcass (Location); whether within two hours of sunrise/sunset, day, or night (Diel), or whether timing was soon after release (PostRelease). The model with the best fit is selected as the one with the lowest AIC value (delta = 0).

Model description	df	logLik	AIC	delta	weight
Location	4	-201.8	411.7	0	0.809
Location + Diel	6	-201.6	415.3	3.6	0.135
Global	7	-201.5	417	5.4	0.056
PostRelease	2	-215.8	435.7	24	0
null	2	-215.8	435.7	24	0
Diel	4	-215.4	438.8	27.2	0
Diel + PostRelease	5	-215	440	28.3	0

## Discussion

The footage collected on video collars carried by devils in coastal northwest Tasmania, Australia, provide the first quantification of foraging behaviour and social interactions throughout all aspects of daily life, a goal that has been previously unattainable using existing methods. Devils are predominately nocturnal and spend most of their time resting at any time of day. Agonistic interactions with other devils occur occasionally and mostly when devils are travelling. Devils at this site scavenge more than they hunt. The video collars documented evidence of killing behaviour by devils for the first time. The two hunting and killing events recorded on the video collars are of small prey animals. They appear to be opportunistic, involving only a short pursuit while the devils were travelling, with no extended approach or pursuit.

The primacy of scavenging over hunting by devils at the Arthur Pieman Conservation Area and adjacent livestock properties indicates that scavenging is an important part of the foraging ecology of this specialist bone-eating carnivore. While all mammalian carnivores scavenge facultatively, albeit to widely differing extents [35], few species have morphological specialisations to deal with the tough parts of carcasses, these species being limited to the placental hyaenas (Family Hyaenidae) [36] and the marsupial analogue, the Tasmanian devil [25]. The devil is closest ecologically to the brown hyaena (*Hyaena brunnae*), scavenging and hunting solitarily but congregating around carcasses [37, 38], rather than the more predatory striped hyaena (*Hyaena hyaena*) [39] or group-living spotted hyaena (*Crocuta crocuta*) [26]. Devils are the only carnivore in Australian ecosystems that can fully utilise carcasses and, as the largest mammalian predator in Tasmanian ecosystems, they can behaviourally exclude other species of scavengers [9, 40]. This may facilitate their energetic as well as numerical dominance of the mammalian carnivore guild [20]. The extent to which devils hunt and scavenge likely depends on the availability of carrion in space and time, as is found for other carnivores [41]. The mixed agriculture and Conservation Area of our study site provides both abundant macropod prey for devils [22] and high availability of carrion, in the form of dead cows and macropods culled under crop protection permits on the livestock farm and roadkill along the sealed road that runs the length of the site. The high density of devils at this study site (Potts et al. Unpublished data) may explain why scavenging records in the video sample were dominated by small items, with few large carcasses encountered, as carrion would be consumed quickly.

The importance of scavenging has implications for trophic cascades within the scavenger community in Tasmanian ecosystems in the light of the severe and sustained decline of the species from devil facial tumour disease. Following 77% decline in devil populations in diseased areas, which now cover most of the devil’s range [5], carrion is persisting much longer in the environment [40]. This increased quantity of carrion is available to other vertebrates and invertebrate scavengers and microbial decomposers. The main beneficiaries among the vertebrate mesopredators are the spotted-tailed quoll (*Dasyurus maculatus*), feral cat and forest raven (*Corvus tasmanicus*) although these smaller species are not functionally equivalent to devils, being unable to compensate for the volume of carrion eaten by devils [40]. Other evidence of these changes is a greater proportion of large prey species in the diet of the spotted-tailed quoll, probably as carrion rather than kills, in eastern Tasmania [22], where DFTD first emerged in the mid-1990s and where devil populations have been at greatly reduced densities for 25 years.

Scavengers are increasingly being recognised as important agents of carrion removal and associated ecosystem services [42, 43]. Consumption of carrion by carnivores such as the golden jackal (*Canis aureus*) and the spotted hyena play an important role in minimising disease risk to livestock and humans [44, 45]. Carrion provides a key food resource for breeding in blowflies (Diptera: Calliphoridae), which have significant economic impacts on livestock health and the price achieved for sheep’s wool [46]. Therefore, we expect devils to perform a useful ecosystem service to livestock farmers as an increased persistence of carrion in the landscape could have a significant economic impact, particularly for wool production.

Video collars provide a new tool for observing a range of natural behaviours in animals, unimpeded by biases from observation. First, video collars allow distinguishing carrion from kills, which is difficult to do from scat remains unless there are blowfly maggots present or odour of rotting meat carries through. Second, video collars also allow direct quantification of diet composition, without the biases of scat analysis [47], although ground vegetation and low lighting can limit prey identification. Third, aspects of behaviour can be directly quantified in a way that has not been previously possible for cryptic, nocturnal species. While distance, habitat, and speed and tortuosity of travel can be quantified from GPS tracking, allowing inferences about foraging behaviours, video collars can reveal the context of scavenging and hunting behaviours. The video collars revealed that when devils are active, they run at a constant pace and rarely walk, and they constantly and opportunistically forage (for carrion or live prey). The majority of scavenging occurs in natural vegetation, although devils also scavenge in pasture and along roads, and hunting was observed in both native vegetation and pasture. Finally, video collars enable new detail of intraspecific interactions.

Video collars enable direct observation and quantification of interactions and biting contacts that occur away from carcasses, the main context where devils can be observed directly [11], or dens that are found using radiotracking and can be monitored using remote cameras. At our study site, where the density and chance of encountering another devil are high, the majority of interactions occurred when the collared devil was traveling, and three-quarters of these involved vocalisations and a brief chase but no physical contact. Interactions at dens and carcasses were much less frequent but with a similar low frequency of physical contact and biting. This suggests that the individual devils on the study area, which occupy overlapping home ranges [27] have established dominance relationships, which also are maintained by displays of non-contact behaviours [10]. Little is known about the ecology of wild devil dens but one study found that individuals use between one and ten dens and change dens every few days [19]. The low rate of injurious contacts we recorded during active travel and at dens and carcasses is similar to direct observations of interactions recorded around carcasses [11] and potentially lower than rates of biting injury during mating interactions indicated by indirect methods—proximity-sensing radiocollars and new injuries recorded when these collared individuals are trapped [17]. It is the injurious mating interactions that are thought to contribute most to transmission [17]. Our study thus contributes new information towards building a holistic understanding of the frequency and context of social contacts between devils that are relevant to transmission of DFTD. This body of information is crucial for interpreting social contact networks relevant to transmission of devil facial tumour disease, to predict long-term epidemic outcome [48] and informing management options [49].

Video collars are a useful tool for observing the behaviour of nocturnal and cryptic species, such as carnivores, through all aspects of daily life. Building video collars sufficiently small and light to fit to a devil enabled quantification of behaviours of wild devils not previously seen using conventional field methods. Combining even small amounts of video collar footage with conventional field methods, such as direct observation, remote cameras, GPS collars and determining diet from scat collection can provide a deeper understanding of ecological aspects, such as foraging ecology, habitat selection and social interactions. Most ecological endeavours will benefit from the increased availability of animal-borne video collars, and the deeper insights they can provide on animal behaviour.
